# Evaluation of School Health Policies and Practices in Brazil and Portugal: Protocol for Mixed Methods Research

**DOI:** 10.2196/87902

**Published:** 2026-04-24

**Authors:** Bruna Hinnah Borges Martins de Freitas, Eliane Tatsch Neves, Manuela Costa Melo, Marília Cordeiro de Sousa, Andrea Moreira Arrué, Mariana Matias Santos, Luciana Pedrosa Leal, Maria Angélica Marcheti, Juliano Bortolini, Maria Aparecida Munhoz Gaíva, Carolina Miguel da Graça Henriques, Maria do Céu Coelho Monteiro Pires, Eva Patrícia da Silva Guilherme Menino

**Affiliations:** 1 Faculty of Nursing, Federal University of Mato Grosso, Cuiabá, Mato Grosso, Brazil; 2 Department of Nursing, Federal University of Santa Maria, Santa Maria, Brazil; 3 Department of Nursing, School of Public Health of the Federal District, Brasilia, Brazil; 4 Department of Nursing, Alfredo Nasser University Center, Goiana, Brazil; 5 Postgraduate Program in Biomedical Engineering (PPGEB)–Federal University of Technology Paraná (UTFPR), Federal Institute of Paraná (IFPR), Curitiba, Brazil; 6 Department of Nursing, Federal University of Pernambuco, Av. Prof. Moraes Rego, 844-900 - Cidade Universitária, Recife - PE, CEP 50670-420, Pernambuco, Brazil; 7 Integrated Institute of Health, Federal University of Mato Grosso do Sul, Campo Grande, Mato Grosso do Sul, Brazil; 8 Department of Statistics, Federal University of Mato Grosso, Cuiabá, Mato Grosso, Brazil; 9 UICISA:E, ciTechcare, School of Health Sciences, Polytechnic University of Leiria, Leiria, Portugal; 10 Portuguese Red Cross School of Health, Lisbon, Portugal; 11 ICBAS School of Medicine and Biomedical Sciences, University of Porto, Porto, Portugal

**Keywords:** protocols, school health services, health plans and programs, program evaluation, adolescent health

## Abstract

**Background:**

School health policies and practices are key components of health promotion for children and adolescents and play a central role in shaping healthy school environments, reducing health inequities, and fostering intersectoral collaboration between education and health systems. Despite their relevance, systematic and comparable assessments of how these policies and practices are implemented across different national contexts remain limited, particularly in low- and middle-income countries. Internationally comparable data are essential to identify strengths, gaps, and priorities for investment in school health.

**Objective:**

The aim of this study is to describe the protocol of a mixed methods study evaluating and comparing school health policies and practices in Brazil and Portugal.

**Methods:**

An explanatory sequential mixed methods design (QUAN → qual → [qual] → [qual]) will be adopted. The quantitative phase (phase I) consists of a cross-sectional survey conducted with school administrators using the Global School Health Policies and Practices Survey, which assesses multiple domains of school health policies, coordination, services, and practices. Quantitative findings will inform the subsequent qualitative phases. Phase II involves semistructured interviews with school principals or head teachers to explore institutional decision-making and policy implementation. Phase III includes interviews with school nurses to examine health service organization, intersectoral collaboration, and professional practices. Phase IV comprises participatory research with adolescents using the photovoice technique to capture youths’ perspectives on school health environments and practices. The study will be conducted in elementary and secondary schools and related health services in selected cities in Brazil and Portugal. Data integration will occur sequentially through connected analyses and joint displays, enabling the development of meta-inferences that link quantitative patterns with qualitative explanations.

**Results:**

The study has secured funding from 2 funding agencies, with project activities initiated in 2025. Quantitative data collection and analysis began in October 2025 in the city of Cuiabá, Brazil. The expansion of data collection to additional Brazilian and Portuguese cities is planned for the first half of 2026. The qualitative phases, including interviews and photovoice activities, are scheduled to take place throughout 2026. The final integrated mixed methods analysis and manuscript preparation are planned for 2027, with dissemination of findings through peer-reviewed journals and national and international scientific conferences by the end of the project cycle.

**Conclusions:**

This study is expected to generate context-sensitive and comparative evidence to support intersectoral actions and inform the development and strengthening of school health promotion policies and practices in different national settings.

## Introduction

### Background

Health actions in schools must be comprehensive and intersectoral, involving families and communities as a shared construction among professionals, services, and schools [1]. In Brazil, the School Health Program (Programa Saúde na Escola—PSE) strengthens promotion, prevention, and care initiatives within Primary Health Care and the Unified Health System through vaccination, oral health, and educational activities [2,3]. In Portugal, the National School Health Program (PNSE) follows the World Health Organization (WHO) health-promoting schools model, integrating health and education through a holistic approach with nurses, psychologists, and community health professionals [4]. In Brazil, actions are mainly carried out through Basic Health Units in a punctual manner, whereas, in Portugal, there is a consolidated structure with school nurses and continuous school health surveillance [5].

Brazil also has the National School Feeding Program (Programa Nacional de Alimentação Escolar), which guarantees daily meals for students and promotes healthy, sustainable eating. In Portugal, schools follow national nutritional guidelines, with menus designed by nutritionists and initiatives to reduce sugar and ultraprocessed food consumption [6]. Physical education is mandatory in both countries: in Brazil, it is reinforced through extracurricular activities and educational campaigns, whereas, in Portugal, it is fully integrated into the pedagogical project, emphasizing active mobility [7].

A comparison shows that, despite structural differences, both countries share a commitment to school health promotion—through programs articulated with the Primary Health Care and the Unified Health System in Brazil and through the consolidation of healthy environments aligned with WHO guidelines in Portugal [8]. However, the Portuguese model stands out for its greater cohesion, advances in mental health, intersectoral coordination, and the prominent role of school nurses [9].

School health promotion has evolved from a biomedical and assistentialist model toward a socioecological approach, which recognizes the school as a complex system and a determinant of children’s and adolescents’ well-being [8]. A review on health-promoting schools in Latin America, however, highlights the scarcity of interventions evaluated with robust methodologies, the absence of large-scale impact indicators, and the need for stronger articulation between science and public policy [10].

These experiences indicate that effective policies depend on intersectoral cooperation, community involvement, and consistent investments in health and education. In this context, it becomes necessary to critically analyze school environments in Brazil and Portugal in light of international guidelines [11,12].

This study describes the protocol of a mixed methods study adopting a complex explanatory sequential design, detailing the planning, data collection, and analysis of school health policies and practices, including the Global School Health Policies and Practices Survey (G-SHPPS) as the central instrument. The synthesis will be conducted collaboratively by Brazilian and Portuguese researchers.

### School Health Policies and Practices

The WHO advocates for salutogenic and health-promoting approaches within the school environment, recognizing the interdependence between health and education as essential to sustainable human development [4]. In this regard, the health-promoting school is conceived as an ecosystem of learning and citizenship, grounded in the values of equity, inclusion, sustainability, democracy, and empowerment, which guide actions toward equal access, appreciation of diversity, and community participation [8].

This model is structured around 6 components: healthy school policies; physical and social environment; individual skills; engagement with the community and school health services; as well as accreditation, continuous evaluation, intersectoral coordination, and qualified professional training [8,13]. The health-promoting school perspective is based on the premise that the quality of education is directly linked to the well-being of the entire school community. WHO and UNESCO (United Nations Educational, Scientific and Cultural Organization) emphasize that no educational system can be effective without promoting the health of students, staff, and families, highlighting key pillars, such as active participation, safe environments, inclusion of health competencies in the curriculum, collaboration with families and communities, and access to school health services [14].

The operationalization of this model requires sustainable intersectoral policies, evidence-based practices, continuous professional development, and a commitment to equity and inclusion. Recognizing the school as a strategic setting for health promotion from early childhood underscores its transformative role, in alignment with the Sustainable Development Goals of the 2030 Agenda [15].

Although most countries have some form of school health services, the WHO guideline on school health services identifies limitations, such as a lack of evidence-based foundations, weaknesses in implementation, underfunding, and limited reach [4], reinforcing the urgency of more robust, integrated, and equitable models.

### Global School Health Policies and Practices Survey

The G-SHPPS is an international tool designed to assess school health policies and practices across 5 main components: healthy and safe school environment, health services, nutrition, health education, and physical education [16]. Its purpose is to support countries in defining priorities, implementing programs, allocating resources, and monitoring trends, thereby enabling comparisons across different contexts.

The global version of the G-SHPPS is derived from the School Health Policies and Practices Study, developed by the Centers for Disease Control and Prevention in the United States since the 1990s [17]. Based on this foundation, the WHO and Centers for Disease Control and Prevention conducted methodological revisions that led to the current global format.

In Brazil, the G-SHPPS was cross-culturally adapted by the researchers of this project to ensure its suitability for the national context. The process included translation, cross-cultural adaptation, and expert committee review, addressing semantic, idiomatic, conceptual, and cultural equivalence. The instrument achieved a total content validity index of 0.99 and demonstrated strong semantic validation among the target audience (school administrators), indicating high agreement among evaluators regarding the relevance and adequacy of the Brazilian version [18].

In Portugal, the cross-cultural adaptation of the G-SHPPS is planned and will be conducted following approval by the relevant ethics committee, using methodological steps equivalent to those adopted in Brazil. These steps will include independent forward translation by bilingual translators familiar with the educational and health contexts, synthesis of translations, and review by an expert committee composed of researchers with expertise in school health, public health, nursing, education, and survey methodology from both countries. Decision rules will be based on predefined equivalence criteria, with items retained, revised, or excluded according to expert consensus and content validity index thresholds.

The original version of the questionnaire is publicly available [16] and represents a valuable tool to support diagnostics, international comparisons, and the strengthening of educational and health policies in both countries.

### Research Questions and Project Objectives

This project was conceived through a partnership between Brazilian and Portuguese researchers, with the overarching goal of evaluating and comparing school health policies and practices in both countries. Based on the data generated through the application of the G-SHPPS instrument with school administrators, sequential interviews by the principal or head teacher (or the person acting in that capacity) and school nurses, interviews with adolescents using the photovoice technique, and the integration of quantitative and qualitative data, the study seeks to address the following research questions: (1) what is the profile of the principal or head teacher of the participating schools? (quantitative); (2) what are the characteristics of school health policies and practices in both contexts, considering school health services, physical environment, food and nutrition services, health education, physical education, school governance and leadership, and school policies and resources? (quantitative); (3) do school health policies and practices differ between schools that have adhered to the Brazilian PSE and those that have not? (quantitative); (4) what are the perceptions of administrators and school nurses in Brazil and Portugal regarding school health policies and practices? (qualitative); (5) what are adolescents’ perceptions of school health policies and practices? (qualitative); (6) to what extent do adolescents participate in the design, implementation, and monitoring of these policies and practices? (qualitative); (7) how do the quantitative results on the characteristics of school health policies and practices converge or diverge from the qualitative perceptions of school administrators and nurses? (mixed methods); (8) which aspects of school health policies and practices, identified quantitatively, are corroborated or complemented by adolescents’ qualitative reports? (mixed methods); and (9) how do quantitative and qualitative data complement each other to identify priorities for investment in school health? (mixed methods).

The specific objectives of this project are: (1) to describe the profile of the principal or head teacher of elementary and secondary schools (quantitative); (2) to characterize existing school health policies and practices in both contexts, considering the domains of school health services, physical environment, food and nutrition services, health education, physical education, school governance and leadership, and school policies and resources (quantitative); (3) to compare school health policies and practices implemented in schools that have adhered to the Brazilian PSE with those that have not (quantitative); (4) to analyze the perceptions of administrators and nurses regarding school health policies and practices (qualitative); (5) to understand adolescents’ critical awareness of school health policies and practices (qualitative); (6) to identify the extent to which adolescents have participated in the design, implementation, and monitoring of school health practices in each country (qualitative); (7) to examine how the quantitative results on the characteristics of school health policies and practices converge or diverge from the qualitative perceptions of school administrators and nurses (mixed methods); (8) to analyze which quantitative aspects of school health policies and practices are corroborated or complemented by adolescents’ qualitative reports (mixed methods); and (9) to develop a list of priorities for investments in school health policies and practices in both countries, based on the integration of quantitative and qualitative findings (mixed methods).

## Methods

### Study Design

This is a mixed methods study adopting a complex explanatory sequential design (QUAN → qual → [qual] → [qual]), as outlined by Creswell and Creswell [19] (2021). This approach was chosen because it allows for an in-depth and contextualized exploration of a phenomenon initially described through quantitative data.

In the quantitative phase (QUAN), a cross-sectional study will be conducted with school administrators using the G-SHPPS instrument (phase I). Subsequently, in the qualitative phases (qual), the initial findings will be further explored through semistructured interviews by the principal or head teacher (or the person acting in that capacity) (phase II) and school nurses (phase III), employing an exploratory-descriptive approach. Phase IV will consist of a participatory study, applying the photovoice technique with adolescent students.

**Figure 1.**
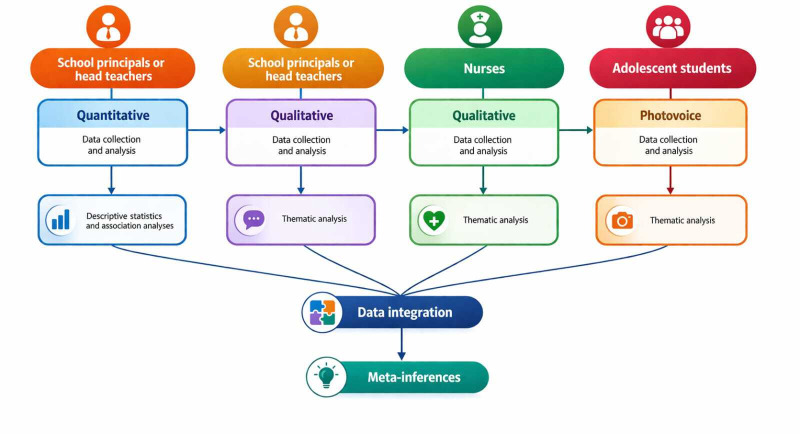
Diagram of the explanatory sequential mixed methods study with a multiphase design.

The phases are organized sequentially, and the data will be connected and integrated at the end of the study, with the aim of producing meta-inferences on school health policies and practices, as well as proposing investment priorities for both the Brazilian and Portuguese contexts. For better visualization, the study design diagram is presented in Figure 1.

### Study Setting

The study will be conducted in elementary and secondary schools, as well as in health services linked to the school territory, located in different cities across Brazil and Portugal. The selection of these settings aimed to represent the diversity of institutional, regional, and intersectoral contexts involved in the implementation of school health policies and practices.

The study settings were selected to reflect the geographical and administrative complexities of both nations:

Brazil: Being a continental-sized country, Brazil presents significant regional, socioeconomic, and cultural heterogeneities. To capture this diversity, the research will take place in 8 cities across different regions: Brasília (DF), Campo Grande (MS), Cuiabá (MT), Curitiba (PR), Goiânia (GO), Recife (PE), Manaus (AM), and Santa Maria (RS). This selection ensures a representative overview of the PNSE implementation across its decentralized territory. Portugal: Although the geographical scale is smaller, the selection of Cascais and Leiria supports the transferability of findings by representing diverse territorial models within Portugal’s centralized system.

Cascais, part of the Lisbon Metropolitan Area, provides insights into school health management in high-density urban environments with well-established intersectoral networks. Leiria represents a vital regional hub that encompasses both urban and semiurban characteristics, a common profile among medium-sized Portuguese cities. Both municipalities feature high maturity in the PNSE, offering a robust basis for comparison against the Brazilian reality.

### Data Collection Environments

Data collection activities will take place in school environments and health services where nurses work in connection with the participating schools. The specific sites will be defined collaboratively with school management teams to ensure privacy and comfort for participants, appropriate conditions for conducting interviews and administering questionnaires, and adequate space for holding participatory workshops.

### Study Population

The study population consists of 3 main groups of participants: principals or head teachers (or the person acting in that capacity), nurses working in the participating schools, and adolescents enrolled in the selected schools. These groups were defined according to the specific objectives of the study and the multiactor perspective that guides the analysis of school health policies and practices.

### Phase I (Quantitative)

#### Participants and Recruitment

Participants in this phase will be school principals or head teachers (or the person acting in that capacity) of public and private elementary and secondary schools. The sampling plan follows the WHO G-SHPPS standardized guidelines to ensure high-quality data and international comparability. A stratified random sampling design will be implemented in both Brazil and Portugal, using official school registries (National Institute for Educational Studies and Research Anísio Teixeira, Brazil and the GesEdu platform, Portugal).

Schools will be stratified by geographic location (participating cities) and school type (public vs private). The sample size was calculated based on an assumed prevalence of 50%, a 95% confidence level, and a margin of error of 5%. To maintain the integrity of the probability sample and prevent imbalances, a systematic nonresponse handling procedure has been established: if a selected school declines to participate or does not respond after 3 contact attempts, it will be replaced by the next randomly selected school from the same stratum in the reserve list.

School administrators from the selected schools will be invited in person through visits conducted by the local research team. Upon agreement to participate, they will receive a link to the informed consent form and the electronic questionnaires to be completed.

#### Quantitative Data Collection

Data will be collected through the application of 2 self-administered instruments: a sociodemographic questionnaire developed by the researchers to characterize the profile of school administrators and the G-SHPPS [16], adapted and validated for the Brazilian and Portuguese contexts.

The G-SHPPS is a high-quality global surveillance system developed by the WHO that combines a standardized scientific sample selection process, a common methodology, and a common self-administered questionnaire. The implementation of this survey is supported by the WHO technical framework, which includes aid in sample design, questionnaire development, and provision of implementation handbooks. Survey coordinators in both countries participate in WHO training workshops to ensure that survey administration procedures are standardized, enabling direct comparability of data between Brazil and Portugal.

The instruments will be made available on an online platform, to be completed individually at the time and place of the participant’s choice. The research team will provide technical support whenever necessary to ensure data quality and completeness. Following WHO guidelines, data will be edited and weighted to adjust for selection probabilities and nonresponse, ensuring that the findings are representative of the target populations.

#### Quantitative Data Analysis

The instruments will be developed in Google Forms, and the data will be organized in spreadsheets for processing. Data analysis will be conducted using IBM SPSS Statistics (version 28.0) and R (version 4.2), software developed by the R Foundation for Statistical Computing.

Descriptive analysis will include frequencies, proportions, means, and SDs to characterize school profiles and participants. To meet the rigor required in surveys, the analysis will follow these criteria:

Primary end points and comparisons: The primary end points consist of the mean scores obtained in the five core domains of the G-SHPPS: (1) school health services, (2) school physical environment, (3) food and nutrition services, (4) health education, and (5) physical education.

Comparison between public and private schools: the type of school administration (public vs private) will be treated as a central independent variable and as a stratum in the sampling design. Advanced multivariate modeling will be used to compare scores between these sectors, allowing for the identification of disparities in policy implementation regardless of the national context.

Clustering adjustment: to account for the hierarchical nature of the data, the statistical design will include adjustments for clustering effects at the city/municipality level. This will ensure correction for intraclass correlation, considering that schools within the same locality may share similar structural characteristics.

Multiple comparisons: due to the analysis of multiple domains and indicators, the Benjamini-Hochberg method will be used to control the false discovery rate reporting q-values to mitigate the risk of type I error (false positives).

Data weighting: statistical weights recommended by the WHO will be applied to adjust selection probabilities and compensate for any nonresponse rates, ensuring that the findings are representative of the target school population.

### Phases II and III (Qualitative)

#### Participants and Recruitment

These phases will include two groups of participants: (1) the principal or head teacher who participated in the quantitative phase and (2) nurses working in those schools.

In phase II, the principal or head teacher will be purposively selected based on the preliminary analysis of quantitative data and according to criteria of contextual variability (type of school, geographic location, and adherence to the Brazilian PSE).

Initially, 5 principals/head teachers per participating location (total n=50 across the 10 study sites) will be invited to participate in semistructured interviews.

Selection will follow a maximum variation strategy informed by patterns identified in the quantitative findings, including differences in the presence or absence of specific school health policies, diversity of implemented practices, school type (public vs private), geographic context, and program adherence status.

The goal is to include diverse profiles of experience and perception regarding the implementation of school health policies and practices. The findings from phase II will provide an institutional and managerial perspective on how school health policies are interpreted, prioritized, and operationalized at the school level, serving as the first explanatory layer of the sequential design.

In phase III, nurses working in these schools will be invited through direct contact with the research team. Efforts will be made to ensure regional representativeness and diversity of experiences. Similarly, 5 nurses per participating location (total n=50 across the 10 study sites) will initially be invited to participate in semistructured interviews. The number of participants will be determined based on the concept of information power [15].

According to this framework, sample size adequacy depends on the study aim, sample specificity, use of established theory, quality of dialogue, and analytic strategy. Given the focused research aim, participant specificity, and explanatory sequential design, the initial estimate of 5 participants per professional group per location is considered methodologically appropriate. However, the final sample size may be adjusted according to the principle of information power to ensure sufficient analytical depth [15].

#### Qualitative Data Collection

Phases II and III will employ individual semistructured interviews, conducted either in person or remotely, depending on participants’ availability. The interview guides will be developed based on the quantitative findings and organized around thematic axes aligned with the study objectives.

The findings from phase I (quantitative) will guide the development of both phases II and III interview guides, focusing on policy and practice patterns that require contextual interpretation.

Interviews with the principal or head teacher (phase II) will aim to deepen the understanding of school health policies and practices from a managerial perspective, exploring how these patterns are interpreted, prioritized, and operationalized at the institutional level. Interviews with nurses (phase III) will explore how these same policy and practice patterns are perceived, negotiated, and implemented in daily professional practice, allowing comparison and contrast between managerial and professional perspectives.

All interviews will be audio-recorded and subsequently transcribed verbatim.

#### Qualitative Data Analysis

Qualitative data will be analyzed using reflexive thematic analysis as proposed by Braun and Clarke [20] (2006), aiming for immersion and deep engagement with the data [21].

The analysis will be conducted by 2 researchers to ensure reliability and research integrity, following the 6 steps recommended by Souza [21] (2019). Initially, data will be fully transcribed, reviewed, and repeatedly read for in-depth familiarization, seeking meanings and patterns through a deductive approach, with preliminary coding ideas noted during this process.

Next, initial codes will be generated deductively from the data, guided by the findings from the quantitative phase (phase I), while maintaining analytical sensitivity to distinctions between managerial (phase II) and professional practice (phase III) perspectives.

Subsequently, codes will be organized into potential themes by grouping relevant extracts and examining how they interrelate to form broader categories or subthemes. These themes will then be reviewed and refined to ensure internal consistency and clear distinctions between them.

In the following stage, themes will be clearly defined and named according to their core meaning and relevance to the research questions, with subthemes identified when necessary to structure more complex analytical categories. Finally, a concise and coherent analytical narrative will be developed based on the identified themes and subthemes.

To ensure anonymity, participants will be randomly coded: “P” for principals, “H” for head teacher, and “N” for nurses, followed by the interview number.

### Phase IV (Photovoice)

#### Participants and Recruitment

This phase will be conducted with adolescent students aged 12 to 18 years, who are regularly enrolled in the schools participating in the quantitative phase and who have attended the school for at least 1 year.

Participant selection will be conducted through convenience sampling, based on recommendations from school administrators, while ensuring diversity in terms of gender, age group, and type of school. Adolescents will be recruited specifically from the same schools in which principals/head teachers participated in phase II, ensuring alignment between managerial perspectives and students’ lived experiences within the same institutional contexts.

To minimize selection bias, school administrators will be instructed to nominate adolescents with heterogeneous profiles, explicitly including students with different levels of academic performance, school engagement, and social backgrounds, rather than only those with high visibility or leadership roles.

Whenever possible, additional strategies will be adopted, such as open invitations presented in classrooms or school meetings and consultation with school support staff (eg, counselors or social workers), to broaden access and facilitate the inclusion of adolescents from potentially marginalized or underrepresented groups.

Initially, approximately 5 adolescents per participating location (total n=50 across the 10 study sites) will be invited to participate in the photovoice activities. Given the participatory and context-specific nature of the photovoice methodology, the initial estimate of 5 adolescents per location is considered appropriate, with final inclusion guided by information power to ensure sufficient analytical depth [15].

The selection and focus of this phase will be informed by key findings from phases II and III, particularly those related to discrepancies between institutional intentions, professional practices, and adolescents’ lived experiences. Providing a standardized camera to all participants aims to reduce socioeconomic barriers and further support inclusive participation.

#### Qualitative Data Collection

Adolescents will be provided with an instant-print camera by the research team for image production, eliminating the need for personal devices and ensuring equitable participation. The photovoice technique will be applied, a participatory visual methodology developed by Wang and Burris [22] (1997), aimed at enabling adolescents to express their experiences through photography, fostering critical reflection and the potential for social transformation. This approach seeks to promote community engagement, empowerment, and advocacy [23].

The process will include 3 interconnected stages. It will begin with an initial training session, during which adolescents will be introduced to the study objectives, provided with ethical guidance, and instructed on how to capture images.

Following this preparation, participants will engage in the image production stage. Each adolescent will be invited to take photographs that, in their view, represent the presence (or absence) of school health policies and practices in their schools. The records should cover the domains of the G-SHPPS. Participants will be asked to submit up to 2 images per domain, accompanied by descriptive captions addressing the following questions: What is in the image? What type of action or absence does it represent? Where was it taken? The domains emphasized during image production will be guided by critical issues identified in the previous qualitative phases. The findings from phases I, II, and III will inform the thematic focus of image production, ensuring that adolescents are encouraged to reflect on policy patterns, managerial interpretations, and implementation practices previously identified, thereby strengthening the explanatory sequential logic of the overall study design.

In the final stage, the photographs produced will be discussed with adolescents through individual interviews using the SHOWED approach [22], which explores the following questions: What do you See here? What is really Happening? How does this relate to Our lives? Why does this situation Exist? How could this image Educate the community or policymakers? What can we Do about it? Discussions will be audio-recorded and subsequently transcribed verbatim.

#### Qualitative Data Analysis

The qualitative data will be analyzed using inductive thematic analysis as proposed by Braun and Clarke [20] (2006), aiming for immersion and deep engagement with the data [21]. The analysis will be conducted by 2 researchers following the 6 steps previously described. This inductive analysis will constitute the final explanatory layer of the sequential design, allowing adolescents’ perspectives to contextualize, confirm, or challenge interpretations derived from phases II and III.

To ensure anonymity, participants will be randomly coded with the abbreviation “Ad” for adolescents, followed by the interview number.

### Data Integration

The quantitative results from the G-SHPPS will inform the development of qualitative interview guides with school administrators (phase II), school nurses (phase III), and adolescents (phase IV). Upon completion of all phases, data integration will occur through sequential connection and comparison, consistent with the explanatory design. This process will use joint displays [24] to articulate quantitative and qualitative findings across the main axes of analysis—domains of school health policies and practices, differences between schools with and without adherence to the PSE, and levels of adolescent participation. Joint displays will be structured as integrative matrices in which quantitative indicators derived from the G-SHPPS (eg, presence, absence, or frequency of specific policies and practices across domains) are presented alongside corresponding qualitative themes generated from interviews and photovoice narratives.

For example, a joint display may juxtapose quantitative findings, indicating limited implementation of student participation practices with qualitative excerpts from administrators, nurses, and adolescents, which explain institutional constraints, professional challenges, or lived experiences related to participation. Another display may contrast schools with and without PSE adherence, combining quantitative differences in reported policies and practices with qualitative explanations of contextual facilitators or barriers.

Meta-inferences will be developed through integrated interpretation of these displays, systematically examining convergence (corroborating findings), divergence (discordant findings), and complementarity (findings that elaborate or expand the quantitative indicators). The generation of meta-inferences will be iterative and theory-informed, aiming to produce integrated explanations and identify priorities for the improvement of school health policies and practices. The specific structure of joint displays and final meta-inferences will be refined according to the empirical results generated in each phase.

### Rigor

Methodological rigor will be ensured through specific strategies aligned with the mixed methods appraisal tool [25] and the recommendations of Lorenzini et al [26] (2024). In the quantitative phase, rigor will rely on probability sampling, the use of a validated and transculturally adapted instrument, clear eligibility criteria, and appropriate statistical analysis. In the qualitative phase, it will be supported by credibility, transferability, dependability, and confirmability criteria through verbatim transcription, independent coding, participant validation, and researcher triangulation. Overall, rigor will be safeguarded by ensuring the quality and legitimation of inferences, maintaining coherence among objectives, study design, integrated analysis, and meta-inferences, with data integration performed through joint displays to reveal convergences, divergences, and complementarities across findings.

### Use of Artificial Intelligence

ChatGPT (OpenAI, version 5.2, professional plan) was used solely to support translation, grammatical revision, and linguistic refinement of the manuscript, including narrative sections of the text. No empirical study data, sensitive information, identifiable participant data, adolescent data, or original research results were entered into the tool at any stage.

All outputs generated by the tool were critically reviewed and validated by the authors, who assessed linguistic accuracy, scientific coherence, and methodological appropriateness prior to incorporation into the paper. Full responsibility for the scientific content, interpretations, and conclusions rests entirely with the research team.

### Ethical Considerations

The study was approved by the Research Ethics Committee of the Federal University of Mato Grosso (UFMT), the coordinating institution of the project, on April 15, 2025 (approval number 7.509.968; Certificate of Presentation for Ethical Review: 77984223.0.1001.8124). Subprojects at participating centers will be submitted to local ethics committees when required, prior to data collection.

Participation will be voluntary and based on informed consent. School principals, head teachers, coordinators, and school nurses will provide written informed consent. For adolescents, written informed assent will be obtained together with consent from parents or legal guardians.

Quantitative data will be collected using Google Forms configured with restricted access and disabled public sharing. Limited identifiable information (school name, participant role, and institutional email address) will be collected solely to identify participating schools and enable recruitment for subsequent qualitative phases. Access to identifiable data will be restricted to the project coordinator and stored separately from analytical datasets. All identifiers will be removed prior to data analysis and dissemination. After the quantitative data collection phase, datasets will be downloaded to secure institutional storage and permanently deleted from the online platform. Participant confidentiality will be preserved throughout the study.

All qualitative data, including interview transcripts and photovoice narratives, will be anonymized prior to analysis. Identifiable information will be removed during transcription, and participants will be identified using role-based alphanumeric codes. The linkage key between identifiers and codes will be stored separately with restricted access and destroyed after completion of data integration.

For the photovoice phase, additional ethical safeguards will be implemented due to the potential inclusion of identifiable visual content. Images will be reviewed by the research team prior to analysis, and photographs containing identifiable individuals will be digitally modified (eg, through face blurring) or excluded, as appropriate, to ensure confidentiality. All images will be stored in secure, password-protected institutional repositories and anonymized prior to analysis and dissemination. All data collected during this phase will be handled with strict confidentiality and used exclusively for research purposes, with access restricted to authorized members of the research team. No identifiable information will be disclosed in any publications or reports. Participants will not receive any financial or material compensation for their participation in this study. Participation will be entirely voluntary, and participants may withdraw at any time without any consequences.

## Results

This binational study is funded by 2 funding agencies, with project activities initiated in 2025, and was designed to generate comparable evidence on school health policies and practices in Brazil and Portugal. The quantitative phase began in October 2025 in Brazil, with initial data collection conducted in the city of Cuiabá and expansion to additional Brazilian cities planned for the first half of 2026, in accordance with the project timeline.

The quantitative data will enable the characterization of school health policies and practices across multiple domains and support comparative analyses between national contexts, school types, and institutional arrangements. These findings will inform purposive sampling and guide the subsequent qualitative phases in both countries.

The qualitative phases are planned to take place throughout 2026 and include interviews with school administrators, interviews with school nurses, and participatory research with adolescents using the photovoice technique, ensuring analytical comparability between Brazil and Portugal.

In Portugal, data collection is planned to take place after completion of the cross-cultural adaptation of the instrument, scheduled for the first half of 2026, following the same methodological procedures and analytical criteria applied in Brazil. The adaptation process will ensure semantic, idiomatic, experiential, and conceptual equivalence between versions, in accordance with established cross-cultural adaptation guidelines. Cross-country comparisons will be conducted only after confirming these equivalence criteria and ensuring that items are interpreted consistently in both contexts. If any relevant discrepancies are identified during the adaptation process, findings will be reported separately to avoid misinterpretation of country-level differences.

The integration of quantitative and qualitative data is scheduled for 2027, using connected analyses and joint displays, allowing the development of meta-inferences that link observed quantitative patterns with contextual explanations derived from both countries.

Study outputs will be synthesized into technical reports, practice-oriented recommendations for policymakers and practitioners, and comparative scientific manuscripts. Submission of manuscripts to national and international peer-reviewed journals and presentation of findings at scientific conferences are planned throughout 2027, in line with the project timeline.

## Discussion

### Principal Considerations

This protocol outlines a mixed methods study to evaluate school health policies and practices in Brazil and Portugal. The integration of quantitative and qualitative data will enable a comprehensive and comparable assessment across contexts. Findings are expected to inform policy and support future research in child and adolescent health.

### Expected Contributions and Implications

This protocol is expected to generate robust and comparative evidence on school health policies and practices in Brazil and Portugal, anticipating the identification of both shared patterns and context-specific gaps across different educational and health system settings. The central hypothesis underpinning this study is that variations in governance, intersectoral coordination, resource allocation, and adolescent participation will be observed between and within countries, influencing the organization, implementation, and perceived effectiveness of school health policies. By integrating quantitative findings from the G-SHPPS with qualitative insights from school administrators, nurses, and adolescents, the study is designed to produce a comprehensive and nuanced understanding of how school health policies are operationalized in real-world contexts.

This protocol is aligned with the international guidelines of the Global Strategy for Women’s, Children’s and Adolescents’ Health (2016‐2030) and the AA-HA! (Global Accelerated Action for the Health of Adolescents) guide [11,12], which emphasize adolescence as a critical life stage and schools as strategic settings for health promotion. Considering that schools play a central role in fostering holistic development, the use of robust, standardized, and contextually adapted instruments is essential for monitoring and evaluating health-promoting environments [4]. In this regard, the articulation between global frameworks and locally contextualized evidence generated through the G-SHPPS reinforces the importance of public policies grounded in empirical data and tailored to specific national and local contexts [16]. Applying the G-SHPPS in both Brazil and Portugal addresses a significant gap in the literature by enabling, for the first time, a standardized and comparative assessment of school health policies across these 2 countries.

From a methodological perspective, the study advances the field by adopting an explanatory sequential multiphase mixed methods design, which allows quantitative results to guide successive qualitative inquiry. This approach strengthens interpretation by integrating structural data on policies and practices with the lived experiences and perceptions of key stakeholders, including school administrators, nurses, and adolescents [27]. The inclusion of photovoice further enhances methodological innovation by foregrounding adolescents’ voices through participatory visual methods, capturing dimensions of school health that are often overlooked in traditional survey-based research [27]. This participatory component is expected to generate actionable insights regarding adolescents’ needs, expectations, and priorities in relation to healthier, safer, and more inclusive school environments.

Despite its strengths, this study may face limitations related to sociocultural, educational, and socioeconomic differences between Brazil and Portugal, which may influence how policies are implemented and perceived. Additionally, contextual heterogeneity within countries may affect comparability across regions and school types. To mitigate these limitations, the study employs a standardized instrument adapted through rigorous cross-cultural procedures, harmonized inclusion criteria, and analytic strategies that consider contextual and socioeconomic variability. The mixed methods design itself enhances analytical robustness by allowing convergence and complementarity across data sources.

Looking ahead, this protocol establishes a foundation for future comparative and longitudinal research on school health policies, including potential expansion to other countries and educational contexts. The findings are expected to inform evidence-based decision-making, support intersectoral collaboration between health and education systems, and guide strategic investment priorities. Dissemination plans include scientific publications, technical reports, presentations at national and international conferences, and knowledge translation products tailored to policymakers, educators, health professionals, and school communities. Collectively, the study aims to contribute to the advancement of health-promoting schools and to the development of more equitable and effective school health policies in both Brazil and Portugal.

### Conclusion

This protocol defines a comprehensive and methodologically robust framework for a binational mixed methods study on school health policies and practices in Brazil and Portugal. By adopting a comparative and integrative approach, the study is positioned to generate evidence capable of identifying strengths, gaps, and context-specific challenges in school health systems, while supporting collaboration between the health and education sectors. The findings derived from this protocol are expected to inform the development, monitoring, and refinement of school health policies in both countries. In addition, the methodological framework proposed here may support future research and interventions in school health and serve as a reference for comparative studies in other national and international contexts.

## Supplementary material

10.2196/87902 Peer Review Report 1 Peer-review report by the National Council for Scientific and Technological Development (CNPq) – Department of Science and Technology (DCIT), Brazil

## Abbreviations

AA-HA!: Global Accelerated Action for the Health of Adolescents
G-SHPPS: Global School Health Policies and Practices Survey
PNSE: National School Health Program
PSE: School Health Program (Programa Saúde na Escola)
UNESCO: United Nations Educational, Scientific and Cultural Organization
WHO: World Health Organization

## References

[1] Neves ETN, Menino E, Arrué AM (2022). Doença Crônica Em Crianças e Adolescentes: Produção de Saberes e Desafios Para a Saúde Coletiva [Book in Portuguese].

[2] (2007). Programa saúde na escola. https://www.planalto.gov.br/ccivil_03/_ato2007-2010/2007/decreto/d6286.htm.

[3] Wachs LS, Facchini LA, Thumé E, Tomasi E, Fassa MEG, Fassa AG (2022). Avaliação da implementação do Programa Saúde na Escola do Programa de Melhoria do Acesso e da Qualidade da Atenção Básica: 2012, 2014 e 2018 [Article in Portuguese]. Cad Saúde Pública.

[4] (2021). WHO guideline on school health services. https://iris.who.int/server/api/core/bitstreams/f99b6820-3a0d-46ae-8b1b-97e59d6d4b06/content.

[5] (2015). Programa nacional de saúde escolar [Report in Portuguese]. https://platform.who.int/docs/default-source/mca-documents/policy-documents/plan-strategy/PRT-AD-17-03-PLAN-STRATEGY-2015-prt-Programa-Nacional-Saude-Escolar-2015.pdf.

[6] (2018). Orientações sobre ementas e refeitórios escolares [Report in Portuguese]. https://www.cm-grandola.pt/cmgrandola/uploads/document/file/8084/anexo_a_orientacoes_ementas_escolares.pdf.

[7] (2021). Programa estratégico do desporto escolar 2021–2025 [Report in Portuguese]. https://www.aesc.edu.pt/desporto/2021-2022/Desporto%20Escolar_programa_estrategico_de_2021_2025.pdf.

[8] Vilaça T, Darlington E, Velasco MJM, Martinis O, Masson J (2019). SHE manual para escolas 2.0: Um Guia Metodológico para Escolas Promotoras de Saúde [Report in Portuguese]. https://www.schoolsforhealth.org/sites/default/files/editor/health-promoting-school/portuguese-she-school-manual2.pdf.

[9] Pereira A, Escola J, Rodrigues V, Almeida C (2020). Parents’ perspectives on the health education provided by clinicians in Portuguese pediatric hospitals and primary care for children aged 1 to 10 years. Int J Environ Res Public Health.

[10] Bastos PO, Cavalcante ASP, Pereira WMG (2020). Health promoting school interventions in Latin America: a systematic review protocol on the dimensions of the RE-AIM framework. Int J Environ Res Public Health.

[11] (2015). Global Strategy for Women’s, Children’s and Adolescents’ Health (2016–2030). https://www.unicef.org/zambia/media/5886/file/Global-Strategy-for-Womens-Childrens-and-Adolescents-Health-2016%E2%80%932030.pdf.

[12] (2018). Global accelerated action for the health of adolescents (AA-HA!): guidance to support country implementation [Report in Spanish]. https://portaldeboaspraticas.iff.fiocruz.br/wp-content/uploads/2021/03/9789275719985-por.pdf.

[13] Silva M da, Almeida A de, Machado JC (2019). Processo de Acreditação das Escolas Promotoras de Saúde em âmbito mundial: revisão sistemática [Article in Portuguese]. Ciênc saúde coletiva.

[14] (2021). Making every school a health-promoting school: implementation guidance. https://iris.who.int/server/api/core/bitstreams/9c1b7efb-3f80-45c5-bbb1-792a9b733907/content.

[15] Malterud K, Siersma VD, Guassora AD (2016). Sample size in qualitative interview studies: guided by information power. Qual Health Res.

[16] Global school health policies and practices survey. World Health Organization.

[17] (2017). School health index: a self-assessment and planning guide. Middle school/high school version. https://www.cdc.gov/assessing-improving-school-health/media/pdfs/Middle-High-Total-2017.pdf.

[18] de Freitas B, Silva NA, Nascimento R da C (2025). Brazilian version of the global school health policies and practices survey. J Sch Nurs.

[19] Creswell JW, Creswell JD (2021). Projeto de Pesquisa Métodos Qualitativo, Quantitativo e Misto [Book in Portuguese].

[20] Braun V, Clarke V (2006). Using thematic analysis in psychology. Qual Res Psychol.

[21] Souza LK (2019). Pesquisa com análise qualitativa de dados: conhecendo a Análise Temática. Arq Bras Psicol.

[22] Wang C, Burris MA (1997). Photovoice: concept, methodology, and use for participatory needs assessment. Health Educ Behav.

[23] Catalani C, Minkler M (2010). Photovoice: a review of the literature in health and public health. Health Educ Behav.

[24] Guetterman TC, Fàbregues S, Sakakibara R (2021). Palgrave Handbook of Mixed Methods for Systematic Reviews.

[25] Hong QN (2018). Mixed methods appraisal tool (MMAT) version 2018: user guide. http://mixedmethodsappraisaltoolpublic.pbworks.com/w/file/fetch/127916259/MMAT_2018_criteria-manual_2018-08-01_ENG.pdf.

[26] Lorenzini E, Osorio-Galeano SP, Schmidt CR (2024). Practical guide to achieve rigor and data integration in mixed methods research. Investig Educ Enferm.

[27] Wasti SP, Simkhada P, van Teijlingen ER, Sathian B, Banerjee I (2022). The growing importance of mixed-methods research in health. Nepal J Epidemiol.

