# Supplementary material for: Evaluation of School Health Policies and Practices in Brazil and Portugal: Protocol for Mixed Methods Research
Source: JMIR Res Protoc. 2026 Apr 24;15:e87902. doi: 10.2196/87902 (PMC13108841; doi:10.2196/87902)
Supplement: Peer Review Report 1 [file resprot-v15-e87902-s001.pdf]

Resultado Final

Identificação da Proposta

Número do Processo: 404482/2025-2

Solicitante: Bruna Hinnah Borges Martins de Freitas

Chamada: UNIVERSAL2024

Título do Projeto: Políticas e Práticas de Saúde Escolar: um estudo com o Global School Health Policy and Practices Survey (G-SHPPS)

Parecer de Deliberação Final

Nota Final

Nota	Ordem	Prioridade
8,27		P040

Resultado da Avaliação

Favorável

Justificativa:

A proposta foi recomendada pelo Comitê de Assessoramento quanto ao mérito técnico-científico. Porém, na análise comparativa entre as propostas desta demanda, não obteve prioridade que permitisse a sua aprovação, considerando a nota final obtida e os recursos orçamentários disponíveis para a Chamada 44/2024. Proposta aprovada após a fase de recursos.

Recursos

Capital	Custeio	Bolsa	Valor Total
R$ 15.000,00	R$ 99.233,70	R$ 84.000,00	R$ 198.233,70

Data de Emissão

Data de Emissão do Parecer: 13/11/2025

Parecer de Deliberação final antes do período recursal

Critério: Originalidade da proposta e relevância do projeto para o desenvolvimento científico, tecnológico e de inovação do País, incluindo potenciais impactos econômicos, sociais e ambientais.

Peso: 3.0 **Nota:** 8.7

Critério: Consistência da proposta considerando a adequação da metodologia, do cronograma e do orçamento aos objetivos, atividades e metas propostos.

Peso: 2.0 **Nota:** 7.8

Critério: Coerência e adequação entre a capacitação e a experiência da equipe do projeto aos objetivos, atividades e metas propostos.

Peso: 2.0 **Nota:** 8.5

Critério: Produção científica ou tecnológica, experiência no gerenciamento de projetos e formação de recursos humanos do proponente na área do projeto de pesquisa.

Peso: 1.0 **Nota:** 6.5

Critério: Plano para Promoção, Popularização e Divulgação Científica e Tecnológica.

Peso: 1.0 **Nota:** 9.0

Critério: Colaboração e parcerias institucionais do projeto (além dos requisitos previstos no item 1.2).

Peso: 1.0 **Nota:** 8.5

Nota Final

Nota	Ordem	Prioridade

Resultado da Avaliação

Desfavorável

Justificativa:

A proposta foi recomendada pelo Comitê de Assessoramento quanto ao mérito técnico-científico. Porém, na análise comparativa entre as propostas desta demanda, não obteve prioridade que permitisse a sua aprovação, considerando a nota final obtida e os recursos orçamentários disponíveis para a Chamada 44/2024.

Data de Emissão

Data de Emissão do Parecer: 28/08/2025

Parecer de Pré-seleção

Critério: A instituição executora é uma ICT?

SIM

Critério: Há membro de equipe deste projeto participando como membro de equipe de outra proposta desta Chamada?

NÃO

Critério: O proponente possui título de doutor?

SIM

Critério: O proponente possui vinculo formal com a instituição de execução do projeto?

SIM

Critério: O proponente possui, na data de término de submissão, qualquer inadimplência com o CNPq e com as Administrações Públicas Federal, diretas ou indiretas?

NÃO

Critério: A proposta se enquadra na faixa em que foi submetida?
- Faixa A: mínimo de 3 doutores, coordenador não bolsista PQ/DT;
- Faixa B: mínimo de 5 doutores, de ao menos duas instituições nacionais distintas.

SIM

Nota Final

Nota	Ordem	Prioridade

Resultado da Avaliação

Enquadrado

Justificativa:

A proposta se enquadra aos critérios estabelecidos na Chamada.

Data de Emissão

Data de Emissão do Parecer: 27/06/2025

Parecer de Recomendação

Critério: Originalidade da proposta e relevância do projeto para o desenvolvimento científico, tecnológico e de inovação do País, incluindo potenciais impactos econômicos, sociais e ambientais.

Peso: 3.0 **Nota:** 8.7

Critério: Consistência da proposta considerando a adequação da metodologia, do cronograma e do orçamento aos objetivos, atividades e metas propostos.

Peso: 2.0 **Nota:** 7.8

Critério: Coerência e adequação entre a capacitação e a experiência da equipe do projeto aos objetivos, atividades e metas propostos.

Peso: 2.0 **Nota:** 8.5

Critério: Produção científica ou tecnológica, experiência no gerenciamento de projetos e formação de recursos humanos do proponente na área do projeto de pesquisa.

Peso: 1.0 **Nota:** 6.5

Critério: Plano para Promoção, Popularização e Divulgação Científica e Tecnológica.

Peso: 1.0 **Nota:** 9.0

Critério: Colaboração e parcerias institucionais do projeto (além dos requisitos previstos no item 1.2).

Peso: 1.0 **Nota:** 8.5

Nota Final

Nota	Ordem	Prioridade

Resultado da Avaliação

Recomendada

Justificativa:

A proposta aborda temática relevante e atual para a prática clínica. A sua originalidade é por apresentar uma abordagem abrangente e comparativa para a adaptação e aplicação do questionário G-SHPPS, instrumento de referência para políticas e práticas de saúde escolar desenvolvido pela OMS. Propõe como objetivo geral a) Adaptar transculturalmente o questionário Global School Health Policies and Practices Survey em português falado no Brasil e em Portugal e b) Analisar e explorar as políticas e práticas de saúde escolar entre o Brasil e Portugal. O potencial impacto é reconhecível no âmbito social, econômico e ambiental, descrevendo e articulando quais as condições para cada um dos impactos. Com relação a consistência da proposta, o estudo será composto por dois delineamentos: metodológico e de métodos mistos explanatório sequencial (QUAN->qual->[qual]->[qual]). O desenho é complexo pois envolve múltiplas etapas, diferentes cenários, com diferentes fontes de coleta de dados e de procedimentos de análise. Os cenários de pesquisa serão 10 instituições escolares de 8 estados brasileiros, representando todas as regiões e 2 de Portugal, também é complexa esta constituição. Os participantes serão diretores/coordenadores e enfermeiros escolares. Descreve cada uma das etapas, no entanto, informações mais precisas sobre a etapa operacional do estudo são necessárias, para todas as etapas, por exemplo, etapas 2 e 3 (número de participantes, critérios de inclusão e exclusão, referencial teórico, teor da entrevista e procedimentos de confiabilidade dos dados, dadas as diferenças regionais, nacional e internacional); na fase 4, não apresenta de forma clara como ocorrerá o grupo focal. Há indicativos da potencial contribuição e aplicabilidade da proposta para a qualidade do cuidado a esta população, embora persistam algumas dúvidas quanto ao arcabouço teórico e metodológico. Faz menção aos procedimentos éticos. O cronograma descreve as diferentes etapas, pela complexidade do projeto é necessário ajustes e acompanhamento rigoroso da proposta. O orçamento inclui itens parcialmente coerentes em termos de natureza do projeto, mas que podem ser reduzidos quantitativamente sem prejuízo para a exequibilidade da proposta, como material de custeio (material de consumo e serviços de terceiros). Ainda, solicita 8 bolsas de iniciação sem justificativa plausível, representando cerca de 45% do valor solicitado, os ajustes também são necessários. A equipe é composta por número suficiente de integrantes, indicando os coordenadores de cada centro, com funções definidas, com qualificação adequada e experiência no tema. A coordenadora é jovem doutora, com experiência nas temáticas atenção primária a saúde infantojuvenil, saúde escolar, saúde de estudantes, família e comunidade, impacto do uso de telas na população infantojuvenil e estudos de métodos mistos. No que diz respeito a contribuição científica, o índice H Scopus é 8 e Wos é 7. Tem um projeto financiado, como coordenador e outro como membro de equipe, na temática em tela ou afins. Na formação de recursos humanos período analisado orientou 6 de iniciação científica, não teve orientações de mestrado e doutorado concluídas. Plano de divulgação amplo, incluindo o entorno acadêmico, gestores, formuladores de políticas públicas e para a população geral, destacando estratégias como criação de site, newsletters, oficinas nas escolas, produção de material instrucional, ações nas mídias e photovoice, contribuindo para a popularização da ciência. A proposta conta com colaboração de grupos de pesquisa de duas universidades internacionais, além das mencionadas como requisito. Pelo exposto, o CA-EF parabeniza a pesquisadora pela submissão da proposta, recomendando-a, com ajustes no orçamento e de acordo com a disponibilidade orçamentária.

Recursos

Capital	Custeio	Bolsa	Valor Total
R$ 15.000,00	R$ 99.233,70	R$ 84.000,00	R$ 198.233,70

Data de Emissão

Data de Emissão do Parecer: 29/05/2025

Parecer de Ad Hoc

Critério: Avalie a originalidade da proposta e relevância do projeto para o desenvolvimento científico, tecnológico e de inovação do País, incluindo potenciais impactos econômicos, sociais e ambientais.

A proposta apresenta originalidade e relevância, tanto do ponto de vista científico quanto social e tecnológico, ao propor a adaptação transcultural do instrumento Global School Health Policies and Practices Survey (G-SHPPS) para os contextos brasileiro e português. Trata-se de um avanço metodológico e técnico inédito no cenário lusófono, uma vez que o G-SHPPS, embora consolidado internacionalmente como referência para avaliação de políticas de saúde escolar, ainda não havia sido devidamente adaptado linguística e culturalmente para aplicação nesses países. A proposta responde, assim, a uma lacuna histórica na produção de dados comparáveis e válidos sobre políticas escolares de saúde, especialmente relevantes diante das particularidades socioeducacionais de Brasil e Portugal. Do ponto de vista científico, o projeto amplia as fronteiras do conhecimento ao empregar uma abordagem de métodos mistos sequenciais (quantitativo → qualitativo) com integração visual (Photovoice), permitindo uma triangulação aprofundada de dados sobre saúde escolar a partir da perspectiva de diferentes atores: gestores escolares, profissionais de saúde e adolescentes. Esse modelo metodológico contribui para o refinamento de avaliações intersetoriais em saúde e educação, áreas tradicionalmente fragmentadas no campo das políticas públicas. O uso do Photovoice, em especial, representa inovação metodológica relevante ao incluir adolescentes como agentes ativos na produção de evidências, respeitando princípios de participação juvenil e protagonismo, com alto potencial de replicação em outros estudos qualitativos na área de enfermagem ou saúde coletiva. Quanto à inovação tecnológica, destaca-se a criação de uma ferramenta validada e adaptada que poderá ser incorporada como instrumento padronizado de monitoramento no Programa Saúde na Escola (PSE) no Brasil e em programas análogos em Portugal. Essa inovação possui aplicabilidade direta na formulação, monitoramento e reformulação de políticas públicas, permitindo decisões baseadas em dados locais e comparativos internacionais, conforme preconiza a OMS. A metodologia, associada a processos formativos e materiais de apoio, potencializa também o uso da ferramenta como tecnologia educacional de apoio à gestão escolar. No que se refere aos impactos econômicos, o projeto poderá contribuir para a racionalização de gastos públicos ao promover a prevenção em saúde por meio de políticas escolares mais eficazes, o que reduz a incidência de agravos evitáveis e, consequentemente, a demanda futura por serviços especializados. Também impacta positivamente a permanência e desempenho escolar, fatores relacionados à produtividade e à formação de capital humano no longo prazo. Os impactos sociais são expressivos, com ênfase na promoção de ambientes escolares saudáveis e inclusivos, no fortalecimento da equidade e no combate a vulnerabilidades sociais e em saúde. A escuta ativa de adolescentes amplia a cidadania e fortalece a cultura da participação, sendo uma estratégia alinhada às agendas internacionais de direitos da criança e do adolescente. Os impactos ambientais, embora indiretos, ainda podem ser relevantes. O projeto incentiva a implementação de práticas escolares saudáveis e sustentáveis, como alimentação consciente e ambientes físicos adequados, o que pode gerar efeitos comportamentais de longo prazo nas práticas individuais e coletivas dos adolescentes. Em suma, a proposta apresenta alto grau de originalidade, com capacidade de gerar impacto estrutural em políticas públicas de saúde e educação, ao mesmo tempo em que contribui com inovação científica e metodológica de alcance internacional. Trata-se de uma iniciativa consistente, com potencial transformador para o campo da saúde escolar no Brasil e em países lusófonos.

Critério: Avalie a consistência da proposta considerando a adequação da metodologia, do cronograma e do orçamento aos objetivos, atividades e metas propostos.

A proposta demonstra alto grau de consistência interna, com clara coerência entre os objetivos propostos, as estratégias metodológicas adotadas, o cronograma de execução e a estrutura orçamentária. Trata-se de uma iniciativa robusta, sustentada por um plano de ação adequado que contempla todas as fases necessárias à realização de um estudo comparativo internacional com foco em políticas de saúde escolar, empregando abordagens quantitativas, qualitativas e visuais. Adequação da Metodologia é rigorosa, articulando diferentes desenhos para responder a múltiplas perguntas de pesquisa. O estudo metodológico para adaptação transcultural do G-SHPPS segue as recomendações internacionais de Beaton et al. (2007), reconhecidamente robustas para processos de validação intercultural, o que reforça a confiabilidade dos dados a serem gerados. A adoção do delineamento de métodos mistos explanatório sequencial permite explorar não apenas o retrato descritivo e comparativo das políticas de saúde escolar em Brasil e Portugal, mas também interpretar os significados atribuídos a essas práticas por gestores, profissionais de saúde e adolescentes. A integração dos dados, por meio da triangulação entre os achados quantitativos (via G-SHPPS) e qualitativos (entrevistas e Photovoice), é uma estratégia metodológica consistente com os objetivos do estudo e agrega robustez analítica ao desenho proposto. Destaca-se ainda o uso do Photovoice como inovação metodológica de caráter participativo, ampliando o escopo das análises qualitativas para incluir as perspectivas dos adolescentes com base em registros visuais. Trata-se de um diferencial que fortalece o compromisso do projeto com a escuta ativa e a valorização dos sujeitos da pesquisa, além de oferecer subsídios únicos para a formulação de políticas mais sensíveis às realidades juvenis. Quanto ao cronograma, este está distribuído ao longo de 36 meses e detalhado de forma clara com início gradual das atividades e alocação adequada das etapas. O tempo destinado à adaptação do instrumento, coleta de dados, análises, integração de resultados, produção de materiais educativos e disseminação científica é compatível com os métodos propostos. Destaca-se que há fluidez entre as etapas, com transições bem definidas entre fases quantitativas e qualitativas. A fase de disseminação, iniciada no 31º mês, contempla não apenas publicações científicas, mas ações voltadas ao público não especializado, o que demonstra atenção à transferência de conhecimento para diferentes públicos. A proposta apresenta um orçamento bem estruturado e compatível com a natureza e abrangência do estudo. Os recursos de custeio (R$ 133.791,00) e capital (R$ 15.000,00) são alocados de forma racional, priorizando atividades essenciais como deslocamentos para coleta de dados e eventos de monitoramento, serviços especializados para análise e transcrição, além de materiais de apoio logístico. O investimento em 8 bolsas de iniciação científica (R$ 134.400,00) reforça o caráter formativo do projeto e contribui para a qualificação de novos pesquisadores, promovendo a integração entre pesquisa, ensino e extensão. Chama atenção, positivamente, a ausência de itens supérfluos ou inflacionados no orçamento. O uso de tecnologias acessíveis e ferramentas já consolidadas, como tablets para coleta de dados e equipamentos de gravação portáteis, reforça a viabilidade econômica e operacional da proposta. A alocação de recursos é proporcional à complexidade metodológica e ao escopo geográfico da pesquisa, que envolve duas nações com diferentes estruturas de gestão escolar e de saúde pública.

Critério: Avalie a coerência e adequação entre a capacitação e a experiência da equipe do projeto aos objetivos, atividades e metas propostos.

A proposta apresenta uma equipe qualificada, multidisciplinar e experiente, cuja composição está alinhada aos objetivos, às atividades e às metas do projeto. A coerência entre a capacitação dos pesquisadores e a complexidade metodológica e temática da proposta é um dos pontos mais sólidos da submissão, conferindo elevada credibilidade e viabilidade à sua execução. A coordenação do projeto, sob responsabilidade da Dra. Bruna Hinnah Borges Martins de Freitas, é conduzida por uma pesquisadora com histórico consistente de atuação em saúde da criança e do adolescente, promoção da saúde escolar, políticas públicas e inquéritos epidemiológicos. Sua experiência prévia em projetos financiados por agências públicas, bem como sua

liderança no Programa de Extensão InterSaúde na Escola (UFMT), reforça sua capacidade de articulação intersetorial e gestão de equipes multiprofissionais, ambos aspectos centrais nesta proposta. A vice-coordenadora, Dra. Maria Aparecida Munhoz Gaíva, é uma pesquisadora reconhecida na área, com inserção em redes nacionais como a Sociedade Brasileira de Enfermeiros Pediatras e histórico de colaboração com o Ministério da Saúde. Sua experiência com implementação de diretrizes em saúde escolar qualifica a equipe para o diálogo institucional necessário à difusão dos achados da pesquisa. A equipe contempla profissionais com atuação destacada nas áreas de enfermagem pediátrica, saúde pública, epidemiologia, nutrição, educação em saúde, saúde sexual e reprodutiva, além de metodologias participativas e estudos com adolescentes. Há também ampla experiência prévia com adaptação transcultural de instrumentos, validação de questionários, pesquisa com métodos mistos e formação de profissionais da saúde e educação, o que é fundamental para a condução das múltiplas etapas previstas. Os perfis dos docentes pesquisadores vinculados às instituições parceiras nacionais (UFMT, UFSM, UFMS, UFPB, UNIFAN, IFPR, FEPECS, ESA/UEA) indicam elevada produção científica na área de interesse e atuação prática consolidada em projetos com populações escolares, incluindo adolescentes com condições crônicas e necessidades especiais, além de articulação com gestores e serviços públicos. A presença de pesquisadores de campo com experiência local nos diferentes estados brasileiros envolvidos (MT, MS, RS, DF, AM, PB, GO, PR) contribui decisivamente para o bom andamento da coleta de dados e das ações de mobilização, respeitando as especificidades de cada território. Em relação as parcerias internacionais há colaboração com professoras de instituições portuguesas, como o Instituto Politécnico de Leiria e a ESSCVP-Lisboa, fortalece a execução no contexto europeu. Destaca-se a atuação de docentes como Dra. Eva Menino, Dra. Carolina Henriques e Mestre Maria do Céu Pires, todas com envolvimento direto em programas de saúde escolar e experiência em projetos com articulação intersetorial entre saúde, educação e proteção social. Além de contribuírem na adaptação do G-SHPPS para o português de Portugal, elas também garantem a viabilidade logística e ética da coleta de dados em escolas lusitanas. Acrescenta-se que a concessão de 8 bolsas de iniciação científica, com acompanhamento de pesquisadores experientes, reflete o compromisso com a formação de novos quadros qualificados na área de saúde escolar. Esta dimensão formativa é coerente com a missão acadêmica da proposta e contribui para a sustentabilidade científica dos temas abordados.

Critério: Avalie a produção científica ou tecnológica, experiência no gerenciamento de projetos e formação de recursos humanos do proponente na área do projeto de pesquisa.

A produção científica, a trajetória na formação de recursos humanos e a experiência em gestão de projetos da Dra. Bruna Hinnah Borges Martins de Freitas ainda não é consolidada, mas evidenciam um perfil qualificado, com forte inserção acadêmica e atuação prática alinhada à temática do projeto: políticas e práticas de saúde escolar, com foco na população adolescente. A pesquisadora possui 48 artigos completos publicados em periódicos, alguns deles em períodicos com alto fator de impacto, refletindo o momento científico que se encontra pois não possui muitas orientações acadêmicas. A formação de recursos humanos evidencia 13 TCCs orientados e 7 iniciações científicas concluídas; 1 dissertação de mestrado e 1 tese de doutorado em andamento (coorientações). Na pós-graduação a docente ainda não possui muita experiência, mas as temáticas dessas orientações convergem com o projeto, incluindo estudos de adaptação de instrumentos, saúde mental de adolescentes, hanseníase em escolares e práticas educativas no contexto escolar. O potencial da pesquisadora em orientar trabalhos metodologicamente diversos e em níveis distintos da formação acadêmica evidencia seu papel na construção de competências técnico científicas em jovens pesquisadores o que evidencia seu potencial na área é pode ser um aspecto diretamente relacionado à sustentabilidade do campo da saúde escolar no Brasil. Destaca-se também que além da produção bibliográfica, possui produção técnica relevante, incluindo pareceres técnicos, relatórios de pesquisa, desenvolvimento de materiais didáticos e instrucionais, cursos de curta duração ministrados, editoração de anais e participação em programas de rádio e TV. Essa diversidade de formatos mostra capacidade de tradução do conhecimento científico para públicos diversos o que pode ser uma competência central em projetos com dimensão social e política, como a proposta em questão.

Critério: Avalie o plano para promoção, popularização e divulgação científica e tecnológica.

O plano de divulgação científica e popularização do conhecimento apresentado no projeto é compatível com o caráter social, formativo e interdisciplinar da proposta. Ele demonstra sensibilidade às necessidades de diferentes públicos, gestores, profissionais da saúde e educação, adolescentes e suas famílias, e articula múltiplas estratégias de disseminação, o que fortalece o impacto e a aplicabilidade dos resultados para além do meio acadêmico. A proposta contempla ações específicas voltadas à comunidade científica, incluindo publicação de artigos científicos em periódicos nacionais e internacionais; apresentações em congressos, simpósios e seminários acadêmicos; elaboração de relatórios técnicos públicos, com sistematização dos resultados e recomendações para gestores escolares e autoridades de saúde. Essas ações garantem o compartilhamento dos achados com a comunidade científica e a retroalimentação de políticas públicas baseadas em evidências. A presença de pesquisadores com experiência editorial e participação frequente em eventos acadêmicos reforça a viabilidade dessa etapa. Já no que se refere a popularização do Conhecimento para o Público Não Especializado o plano contempla criação de um site do projeto, com seções específicas voltadas a gestores, profissionais de saúde, adolescentes e familiares, onde serão disponibilizados conteúdos acessíveis e atualizações das etapas do estudo. Acrescenta-se envio de boletins digitais (newsletters) para redes de escolas e associações de pais, promovendo comunicação contínua sobre saúde escolar. Destaca-se a divulgação em mídias locais e regionais (rádio, jornal e TV), com press releases e entrevistas para ampliar o alcance público das evidências produzidas. O plano também prevê a produção de materiais com linguagem acessível, o que amplia sua eficácia para públicos com diferentes níveis de letramento. A disponibilização dos produtos em ambientes digitais e físicos garante capilaridade, especialmente em regiões com conectividade limitada. Além disso, a integração de adolescentes como participantes ativos na construção do conhecimento não só fortalece a legitimidade do projeto junto ao público jovem, como promove práticas de divulgação científica engajadoras e inclusivas ? alinhadas aos princípios da ciência cidadã e da educação emancipadora.

Critério: Avalie a colaboração e parcerias institucionais do projeto (além dos requisitos previstos no item 1.2 do regulamento da Chamada).

A proposta demonstra alto grau de articulação interinstitucional, com uma rede de colaboração nacional e internacional consistente e alinhada aos objetivos científicos, metodológicos e operacionais do projeto. As parcerias não apenas cumprem os requisitos formais da chamada, mas se destacam pela diversidade, complementaridade técnica e potencial de sustentabilidade das ações, garantindo robustez na execução e impacto ampliado dos resultados. O projeto conta com a participação de instituições públicas e privadas de diferentes regiões do Brasil, o que confere capilaridade territorial e capacidade de adaptação às realidades locais. Dentre as parcerias destacam-se a Universidade Federal de Mato Grosso (UFMT) Instituição executora, com infraestrutura adequada e liderança consolidada na área de saúde da criança e do adolescente. Sedia o Programa de Extensão InterSaúde na Escola, que amplia a interface entre universidade, escolas e serviços de saúde. Já as instituiçoes UFMS, UFSM, UFPB, UNIFAN, UEA, IFPR, FEPECS ? Cada uma dessas organizações atua como centro participante responsável pela execução em sua respectiva localidade,

ampliando o alcance do estudo e garantindo diversidade de contextos escolares. As parcerias envolvem docentes com ampla experiência em inquéritos epidemiológicos, métodos mistos, políticas públicas, práticas educativas, saúde mental, hanseníase, doenças crônicas na infância e adolescência, inclusão escolar e saúde sexual e reprodutiva. Esse arranjo permite a divisão qualificada de tarefas, otimização de recursos e compartilhamento de expertise entre grupos com reconhecida produção científica. A articulação internacional é outro ponto de excelência do projeto. A proposta formaliza cooperação com instituições de Portugal que possuem tradição na interface entre saúde pública e educação: Instituto Politécnico de Leiria (IPLeiria) e Escola Superior de Saúde da Cruz Vermelha Portuguesa (ESSCVP-Lisboa). Além disso, o projeto conta com autorização formal da Organização Mundial da Saúde (OMS) para adaptação do G-SHPPS, o que fortalece o alinhamento da pesquisa com referenciais internacionais, valida sua relevância política e científica e amplia o potencial de replicabilidade dos achados em outros países lusófonos. A partir do projeto verifica-se que as parcerias não se limitam à coautoria científica, mas refletem uma divisão colaborativa de funções operacionais, com centros locais responsáveis por mobilização institucional. A inserção de discentes de graduação e pós-graduação em todas as fases também favorece a sustentabilidade das ações, ao formar novas gerações de pesquisadores e fortalecer a cultura científica no campo da saúde escolar.

Nota Final

Nota	Ordem	Prioridade

Resultado da Avaliação

Recomendada

Justificativa:

Após análise dos elementos que compõem a proposta intitulada ?Adaptação transcultural do G-SHPPS e análise das políticas e práticas de saúde escolar no Brasil e em Portugal?, verifica-se que se trata de um projeto com ampla pertinência social e potencial concreto de impacto em políticas públicas de saúde e educação. Diante disso e da robustez técnico científica, recomendo seu financiamento. Ao propor a adaptação transcultural do G-SHPPS, a equipe oferece uma ferramenta metodológica inédita para o contexto brasileiro e português, o que potencializa a avaliação e o aprimoramento de ações promotoras de saúde no ambiente escolar. O foco no protagonismo adolescente, por meio da técnica do Photovoice, ainda reforça o caráter emancipador e participativo do projeto, alinhado às diretrizes da OMS e às políticas nacionais de juventude. O projeto demonstra consistência entre seus objetivos, metodologia, cronograma e orçamento. A estratégia de métodos mistos explanatório sequencial é adequada à complexidade do objeto de estudo e permite uma leitura abrangente e aprofundada das políticas de saúde escolar. O cronograma está bem distribuído ao longo de 36 meses, com margens operacionais adequadas e etapas interdependentes bem definidas. O orçamento está compatível com as necessidades do estudo e não apresenta excessos ou fragilidades que comprometam sua execução. A Dra. Bruna Hinnah Borges Martins de Freitas, proponente e coordenadora, apesar de ainda não possui uma trajetória na pós-graduação consolidada pois ainda não possui orientações desta natureza, possui trajetória acadêmica e técnica na área da saúde de adolescentes e políticas públicas, com produção científica condizente com seu momento atual enquanto pesquisadora. Percebe-se experiência em gerenciamento de projetos. O projeto apresenta uma rede articulada de colaborações nacionais e internacionais. A autorização da OMS para adaptação do G-SHPPS e a atuação de pesquisadoras portuguesas diretamente envolvidas em políticas escolares reforçam a validade internacional e a aplicabilidade dos achados da pesquisa. A proposta dedica atenção à disseminação dos resultados, tanto no meio científico quanto junto à comunidade escolar. A criação de um site, newsletters, oficinas em escolas, produção de materiais educativos e ações em mídias locais ampliam o alcance do conhecimento produzido, fomentando a apropriação social dos resultados. A valorização das percepções dos adolescentes, com exposição pública do material produzido via Photovoice, contribui para a popularização da ciência de forma sensível, ética e inovadora.

Data de Emissão

Data de Emissão do Parecer: 23/04/2025

Parecer de Ad Hoc

Critério: Avalie a originalidade da proposta e relevância do projeto para o desenvolvimento científico, tecnológico e de inovação do País, incluindo potenciais impactos econômicos, sociais e ambientais.

A proposta tem como objetivo geral adaptar transculturalmente o questionário Global School Health Policies and Practices Survey (G-SHPPS) para o português do Brasil e de Portugal, bem como, analisar e explorar as políticas e práticas de saúde escolar. A proposta apresentada é original e possui relevância científica e social. A análise comparativa entre Brasil e Portugal é um aspecto que merece destaque. Os impactos econômicos, sociais e ambientais descritos incluem: redução de custos de longo prazo com saúde pública, redução do absenteísmo e evasão, transformação do ambiente escolar em um espaço mais saudável, seguro e inclusivo, e promoção do protagonismo juvenil.

Critério: Avalie a consistência da proposta considerando a adequação da metodologia, do cronograma e do orçamento aos objetivos, atividades e metas propostos.

A metodologia proposta mostra-se adequada e devidamente alinhada aos objetivos do estudo. O estudo será desenvolvido em duas etapas principais: (1) adaptação transcultural do G-SHPPS para os idiomas português do Brasil e de Portugal; e (2) estudo de métodos mistos explanatório sequencial. Participarão do estudo diretores/coordenadores e enfermeiros que atuam nas escolas, além de adolescentes que documentarão suas experiências em saúde escolar por meio de fotografias. O cronograma proposto é exequível e condizente com as etapas do projeto. Quanto ao orçamento, recomendo redução no número de bolsas de iniciação científica e/ou no tempo de duração.

Critério: Avalie a coerência e adequação entre a capacitação e a experiência da equipe do projeto aos objetivos, atividades e metas propostos.

A equipe do projeto é composta por pesquisadores de diferentes regiões do pais (sul, centro-oeste, norte e nordeste), apenas não foram incluídos pesquisadores do sudeste. Na equipe, há duas pesquisadoras com experiência científica consolidada, os demais estão em início de carreira apenas com experiência de orientação de iniciação científica e trabalho de conclusão de curso. Em geral, todos apresentam familiaridade com o tema saúde escolar e/ou com o método de pesquisa (adaptação transcultural e métodos mistos).

Critério: Avalie a produção científica ou tecnológica, experiência no gerenciamento de projetos e formação de recursos humanos do proponente na área do projeto de pesquisa.

A proponente concluiu o doutorado em 2022 e não possui experiência na formação de recursos humanos (nível mestrado e doutorado). É coordenadora de um projeto de pesquisa financiado que está em andamento. Produção científica crescente e em periódicos de reconhecido rigor e seletividade científica. Diante do exposto, considero sua capacidade de liderança e coordenação de projetos de pesquisa e gestão de recursos financeiros incipiente porém com potencial de crescimento.

Critério: Avalie o plano para promoção, popularização e divulgação científica e tecnológica.

Dentre as iniciativas de divulgação propostas destacam-se: divulgação em um site sobre as atualizações, materiais educativos e dados acessíveis, com seções específicas para gestores, profissionais de saúde, adolescentes e pais, newsletters trimestrais enviadas a escolas e associações de pais com informações sobre saúde escolar e atualizações do estudo, encontros presenciais e remotos com gestores escolares, campanhas de Conscientização nas Escolas e Comunidades como palestras e oficinas, divulgação em jornais, rádios e canais de TV locais e regionais, elaboração de folhetos, infográficos, podcasts e guias práticos para adolescentes e suas famílias. Diante do exposto, considero que as iniciativas propostas estão adequadas para promover a popularização da ciência.

Critério: Avalie a colaboração e parcerias institucionais do projeto (além dos requisitos previstos no item 1.2 do regulamento da Chamada).

O projeto conta com a parceria de três pesquisadoras de Portugal, todas com experiência em pesquisas na temática. Serão responsáveis pela adaptação do instrumento G-SHPPS para o contexto português, bem como pela realização de estudos comparativos entre Brasil e Portugal. No Brasil, a equipe do projeto é composta por pesquisadores de diferentes regiões do pais (sul, centro-oeste, norte e nordeste), apenas não foram incluídos pesquisadores do sudeste.

Nota Final

Nota	Ordem	Prioridade

Resultado da Avaliação

Recomendada

Justificativa:

Data de Emissão

Data de Emissão do Parecer: 15/04/2025
